# High Performance Predictable Quantum Efficient Detector Based on Induced-Junction Photodiodes Passivated with SiO_2_/SiN_x_

**DOI:** 10.3390/s21237807

**Published:** 2021-11-24

**Authors:** Ozhan Koybasi, Ørnulf Nordseth, Trinh Tran, Marco Povoli, Mauro Rajteri, Carlo Pepe, Eivind Bardalen, Farshid Manoocheri, Anand Summanwar, Mikhail Korpusenko, Michael N. Getz, Per Ohlckers, Erkki Ikonen, Jarle Gran

**Affiliations:** 1 Department of Microsystems and Nanotechnology (MiNaLab), SINTEF Digital, 0314 Oslo, Norway; Marco.Povoli@sintef.no (M.P.); Anand.Summanwar@sintef.no (A.S.); 2 Department of Solar Energy, Institute for Energy Technology (IFE), 2027 Kjeller, Norway; Ornulf.Nordseth@ife.no; 3 Justervesenet, 2027 Kjeller, Norway; ttr@justervesenet.no (T.T.); jag@justervesenet.no (J.G.); 4 Istituto Nazionale di Ricerca Metrologica (INRIM), 10135 Turin, Italy; m.rajteri@inrim.it (M.R.); carlo.pepe@polito.it (C.P.); 5 Department of Electronics and Telecommunications, Politecnico di Torino, 10129 Turin, Italy; 6 Department of Microsystems, University of South-Eastern Norway (USN), 3184 Borre, Norway; eivind.bardalen@usn.no (E.B.); per.ohlckers@usn.no (P.O.); 7 Metrology Research Institute, Aalto University, 02150 Espoo, Finland; farshid.manoocheri@aalto.fi (F.M.); mikhail.korpusenko@aalto.fi (M.K.); erkki.ikonen@aalto.fi (E.I.); 8 Department of Physics, University of Oslo, 0316 Oslo, Norway; m.n.getz@smn.uio.no; 9 VTT MIKES, VTT Technical Research Centre of Finland Ltd., 02150 Espoo, Finland

**Keywords:** silicon photodetector, inversion layer photodiode, induced-junction, surface passivation, PECVD silicon nitride, radiometry, optical power, primary standard, predictable quantum efficiency

## Abstract

We performed a systematic study involving simulation and experimental techniques to develop induced-junction silicon photodetectors passivated with thermally grown SiO_2_ and plasma-enhanced chemical vapor deposited (PECVD) SiN_x_ thin films that show a record high quantum efficiency. We investigated PECVD SiN_x_ passivation and optimized the film deposition conditions to minimize the recombination losses at the silicon–dielectric interface as well as optical losses. Depositions with varied process parameters were carried out on test samples, followed by measurements of minority carrier lifetime, fixed charge density, and optical absorbance and reflectance. Subsequently, the surface recombination velocity, which is the limiting factor for internal quantum deficiency (IQD), was obtained for different film depositions via 2D simulations where the measured effective lifetime, fixed charge density, and substrate parameters were used as input. The quantum deficiency of induced-junction photodiodes that would be fabricated with a surface passivation of given characteristics was then estimated using improved 3D simulation models. A batch of induced-junction photodiodes was fabricated based on the passivation optimizations performed on test samples and predictions of simulations. Photodiodes passivated with PECVD SiN_x_ film as well as with a stack of thermally grown SiO_2_ and PECVD SiN_x_ films were fabricated. The photodiodes were assembled as light-trap detector with 7-reflections and their efficiency was tested with respect to a reference Predictable Quantum Efficient Detector (PQED) of known external quantum deficiency. The preliminary measurement results show that PQEDs based on our improved photodiodes passivated with stack of SiO_2_/SiN_x_ have negligible quantum deficiencies with IQDs down to 1 ppm within 30 ppm measurement uncertainty.

## 1. Introduction

Silicon photodiodes based on inversion layer (induced-junction) have shown great promise for applications as a calibration standard due to their exceptionally high internal quantum efficiency and the predictability of their response with modelling as well as for applications requiring enhanced responsivity at UV and blue wavelengths [1,2,3,4,5,6,7,8,9,10,11]. Such photodiodes rely on natural formation of an inversion layer at the silicon surface due to fixed charges in the dielectric used for surface passivation and as antireflection (AR) layer. An induced p-n junction is thereby formed without externally introducing impurity acceptor or donor atoms by doping methods such as ion-implantation or gas-phase diffusion [1]. This structure, due to the ultra-thin inversion layer, features a very shallow p-n junction as compared with standard silicon photodiodes where the p–n junction is formed by external doping. The recombination losses in the deeply diffused regions of high donor or acceptor concentration are therefore eliminated. In an induced p–n junction, the recombination-generation centers at the silicon-dielectric interface then become the main limiting factor for quantum efficiency besides the reflection and absorption losses in the dielectric. The challenge of developing ultra-high efficiency photodetectors is therefore reduced to the optimization of the dielectric passivation for low surface recombination velocities and optical losses.

The surface recombination can be reduced by two complementary ways: chemical passivation and field-effect passivation [12]. The dangling bonds at the silicon surface form trap states that capture electrons or holes. These dangling bonds can be completed by a suitable surface dielectric coating and/or chemical species such as hydrogen to reduce the density of interface states or capture probability. This is referred to as chemical passivation. The other way to reduce the surface recombination is to reduce the number of one type of carriers at the silicon surface as the recombination processes require the presence of both electrons and holes. This can be achieved by an electric field that penetrates the silicon surface and repels one type of carriers from the surface, which is referred to as field-effect passivation. Such an electric field is generated by the fixed charges in the passivation dielectric. Therefore, a high fixed charge density in the passivation dielectric is essential for achieving a lower surface recombination velocity as well as for the formation of an induced p-n junction by inversion of the silicon surface. A high fixed charge density is also necessary for a linear photo-response with respect to the incident optical power.

The inversion layer photodiodes were invented in 1978 by T E Hansen [1] as p-type photodiodes based on passivation by thermally grown SiO_2_. It is well known that thermal oxidation provides an excellent chemical passivation for silicon surfaces, and effective surface recombination velocities as low as ∼30 cm/s after a post-oxidation anneal in argon and forming gas have been reported [13]. Due to its positive fixed charge, thermally grown SiO_2_ can be used to passivate the surface of p-type silicon in the inversion mode, leading to a surface inversion photodiode. The first predictable quantum efficient detectors (PQED) were demonstrated using this type of inversion-layer photodiodes [2,3,4,5,6]. The main drawback of thermal SiO_2_ passivation is that the fixed charge density in SiO_2_ is limited to ~1 × 10^11^ cm^−2^ for <100> silicon surface orientation and ~4 × 10^11^ cm^−2^ for <111> surface orientation [14], providing relatively low field-effect passivation and photosensitivity linearity for higher optical fluxes. Therefore, improving the performance of PQEDs requires development of inversion-layer photodiodes with alternative passivation material and processes to achieve as high as possible fixed charge density in addition to a good chemical passivation.

Amorphous hydrogenated silicon nitride, a-SiN_x_:H, (referred to as SiN_x_) deposited by low temperature plasma-enhanced chemical vapor deposition (PECVD) is widely used as surface passivation and anti-reflection coating for crystalline silicon solar cells [12,15,16,17,18,19,20,21,22]. PECVD SiN_x_ has been demonstrated to have one order of magnitude higher fixed charge compared with thermal SiO_2_ [18,20,21,22,23]. Due to the hydrogen content of the deposition process, PECVD SiN_x_ can also provide a good chemical passivation, making it an excellent candidate for realization of inversion-layer photodiodes with extremely low internal quantum deficiency (IQD). Moreover, the stoichiometric ratio of SiN_x_ can be varied, which offers another degree of freedom, to tailor its dielectric and optical properties to meet application-specific requirements [18,19,20,22]. SiN_x_ also functions as a humidity barrier to protect the silicon–dielectric interface from the degrading effects of moisture [24], which is an important asset for the stability of inversion-layer photodiodes. A dielectric stack consisting of a PECVD SiN_x_ film deposited on a thin layer of thermally grown SiO_2_ can potentially provide even superior passivation, leveraging both the excellent chemical passivation characteristics of thermal SiO_2_ and excellent field-effect passivation characteristics of PECVD SiN_x_.

In our work, we have conducted a thorough study on the passivation and optical characteristics of different PECVD SiN_x_ films and their stack with thermally grown SiO_2_. We employed 2D simulations to extract the surface recombination velocity and bulk lifetime and then 3D simulations to predict the response of an inversion-layer photodiode that would be fabricated with such passivation. Based on the predictions of the simulations, we have fabricated inversion-layer photodiodes using a passivation process that leads to unprecedently low IQDs and validated the results by testing photodiodes assembled in a light-trap PQED configuration.

## 2. Inversion Layer Photodiode Structure and Operation

**Figure 1:**
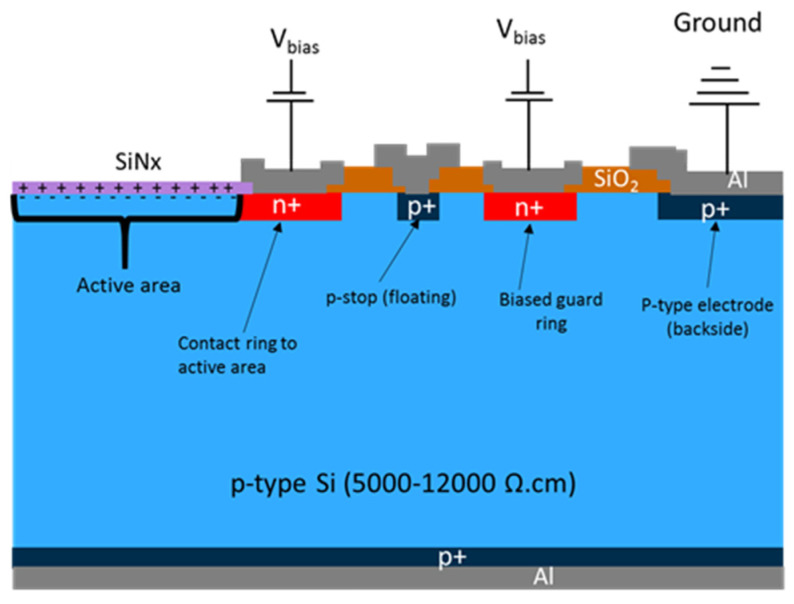
Inversion layer photodiode structure and biasing scheme. The n+ and p+ regions, formed by phosphorous implantation and boron implantation, respectively, have peak doping concentrations of >10^19^ cm^−3^ and profile depths of ~2 µm.

The structure and operation of a p-type inversion-layer photodiode is illustrated in Figure 1. The surface of the active area is inverted by the positive fixed charges in the passivation dielectric. The electrical contact to the active area is made by a heavily phosphorous-doped n+ ring. Another n+ ring is implemented as a guard ring and biased at the same voltage as the active area. Due to the inversion of the surface by the positive field charges in the thermally grown field SiO_2_, the surface outside the active area is also inverted and therefore a p-type inter-electrode isolation structure, namely a p-stop ring, is incorporated into the structure, which remains floating. The physical edge of the photodiode is terminated with p+ doping in order to be able to apply all the biasing from the top side of the photodiode. The photodiode is operated in reverse-biased condition by applying the same positive voltage to the active area ring and the guard ring with respect to the p+ electrode.

## 3. Development of Passivation Process

### 3.1. Methodology

Thanks to improved 2D and 3D simulation models, one can predict the photo response of an inversion layer photodiode made with surface passivation materials of given specific properties. The predictability of response through accurate modelling of losses is not only essential for the use of the photodiodes as a primary standard, but also allows one to optimize the passivation to improve the quantum efficiency of the detector without having to fabricate photodiodes with all possible variations of passivation. The relevant material properties are measured on passivation material grown or deposited on dummy test wafers. The measured effective minority carrier lifetime and fixed charge density are used to extract the surface recombination velocity (*SRV*), which is the limiting factor for internal quantum deficiency, via 2D simulation models. The *SRV* and measured optical properties are then used to predict through 3D simulation models the response of a PQED of inversion-layer photodiodes that would be made with such a passivation material and process. This is an extremely powerful and efficient method that allows one to maximize the quantum efficiency of a PQED through optimization of passivation material and process on dummy test wafers in a time- and cost-effective manner.

In our study, we focused on PECVD SiN_x_ films and their stack with a thin layer of thermally grown SiO_2_ to develop a material system and process that can provide excellent surface passivation for inversion photodiodes with minimal optical absorption and reflection losses. Since the photodiodes will be assembled into a PQED with a 7-reflection light-trap configuration to minimize reflection losses, the optimization of the optical characteristics of the passivation layer is performed accordingly.

#### 3.1.1. Lifetime Measurements

The effective minority carrier lifetime, or simply effective lifetime, ($\tau_{eff}$), in a semiconductor contains contributions from both bulk and surface recombination, and can be expressed as:

(1) $$\frac{1}{\tau_{eff}}=\frac{1}{\tau_{bulk}}+\frac{1}{\tau_{surf}}$$

The lifetime measurements were carried out on double-side-polished, high resistivity, p-type, float zone (FZ) silicon wafers passivated identically on both sides. Such test wafers, which have a long bulk lifetime (typically in the order of 10 ms), are chosen so that the measured effective lifetime is dominated by surface recombination.

For measurement of injection-dependent lifetime curves, the quasi-steady-state photoconductance (QSSPC) method was adopted, using a Sinton WCT-120TS lifetime tester [25]. With this setup, the excess carrier density is calculated from the conductivity of the passivated silicon wafer under illumination, as measured by an inductively coupled coil.

A BT Imaging LIS-R1 unit with an excitation wavelength of 808 nm was used for recording photoluminescence (PL) images of 6-inch surface passivated silicon wafers. The PL intensity was calibrated to the effective minority carrier lifetime based on a QSSPC measurement carried out in the central region of the wafer.

#### 3.1.2. Fixed Charge Measurements

The fixed charge is determined by capacitance—voltage (C—V) measurements on metal-insulator-semiconductor (MIS) structures made on 6-inch, high resistivity, p-type FZ silicon wafers. The measurements were carried out in TSK A-PM-90A automatic probe station using an HP 4284A LCR meter. The AC voltage used in the CV measurements is 100 mV.

The MIS structures are fabricated on a 6-inch wafer and the wafer includes ~120 circular MIS capacitors of an area of 0.035 cm^2^, which also gives a good picture of the uniformity of the film properties across the wafer. The large amount of data obtained from each measurement is converted and sorted using a Python script. The script then also calculates the parameters of interest including dielectric thickness (*t_d_*), flat-band voltage (*V_fb_*), and fixed charge density (*Q_f_*). The flat-band voltage is calculated automatically with an iterative procedure and linear interpolation. The fixed charge is then obtained by:

(2) $$Q_{f}=\frac{C_{acc}}{A\;e}\left|\left(\varphi_{ms}-V_{fb}\right)\right|\;$$

where *C_acc_* is the capacitance in the accumulation mode, *A* is the area of the capacitor, *e* is the elementary charge, and $\varphi_{ms}$ is the metal–semiconductor work function. The high resistivity silicon substrate adds a series resistance, retarding the charging of the capacitor, which results in a measured *C_acc_* lower than the theoretical value. The accumulation capacitance should be equal to the dielectric capacitance, which is given by *C_d_* = *Aϵ_d_*/*t_d_*, where *ϵ_d_* is the permittivity of the dielectric. For improving the accuracy of the results, the measurements were done at low frequencies, making sure that the measured *C_acc_* is reasonably close to *C_d_* which can be calculated by obtaining the dielectric thickness and permittivity from ellipsometry measurements. Hysteresis measurements were also done to make sure that the measured charge is primarily due to fixed charges and that the mobile charge contribution is negligible.

#### 3.1.3. Ellipsometry Measurements and Reflectance Simulations

Thickness, refractive index (*n*), and extinction coefficient (*k*) of the passivating dielectric films are obtained with a non-invasive method applying ellipsometry, an optical technique based on the measurement of elliptically polarized light [26]. Ellipsometric data were collected with a variable-angle spectroscopic ellipsometer (VASE) from J.A. Woollam [27]. Measurements were taken from 400 nm to 850 nm with 10 nm steps at five angles around the Brewster angle.

**Figure 2:**
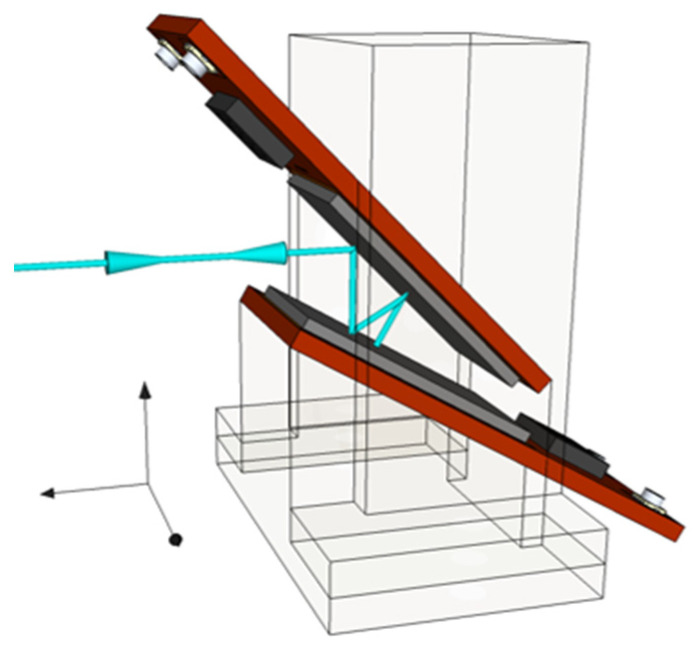
PQED consisting of two photodiodes in light trap configuration.

In order to minimize reflection losses, the PQED assembly consists of two photodiodes mounted in light-trap configuration with an angle of 15° between the diodes, as depicted in Figure 2. In this configuration, the light beam undergoes 7 reflections (one at 0° degree and 2 at 15°, 30°, 45°) following the same incoming and outgoing paths [5,28,29]. Using the results of the ellipsometry measurements performed on passivated test wafers, the reflectance for this PQED configuration was simulated as a function of wavelength for different dielectric thicknesses to determine the optimal thickness for minimum reflectance.

#### 3.1.4. Modelling and Simulations of SRV and IQD

**Figure 3:**
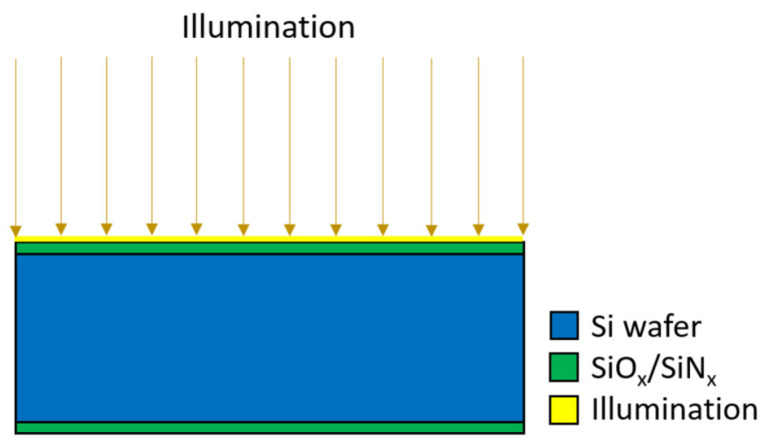
2D lifetime simulation structure.

In order to predict the internal quantum deficiency of photodiode, two-step simulation was performed using the Genius Device Simulator from Cogenda [30]. The first step is to determine bulk lifetime $\tau_{bulk}$ and surface recombination velocity (*SRV*) by using 2D simulation structure and the QSSPC lifetime measurement. This method has previously been presented by Stokkan et al. [31]. The 2D lifetime simulation structure is shown in Figure 3. The structure consists of a 500 µm thick, high resistivity silicon substrate passivated identically on both sides by SiO_2_ and/or SiN_x_ with thickness of two mesh elements. The width of the simulated structure is 7 mm. Excitation light with a wavelength of 808 nm is used for charge carrier generation in the simulation, which is the same wavelength as used in lifetime measurements.

Various parameters from fabrication and tests of samples are used as input for the simulation such as doping type, doping concentration, wafer thickness, fixed charge density *Q_f_*, etc. The effective lifetime $\tau_{eff}$ can be calculated from the effective surface recombination velocity $S_{eff}$ and $\tau_{bulk}\;$ by Equation (1) which can be rewritten as [32]:

(3) $$\frac{1}{\tau_{eff}}=\frac{1}{\tau_{bulk}}+2\frac{S_{eff}}{W}$$

where W is the wafer thickness. The relation between $S_{eff}$ and interface states is described by Shockley–Read–Hall theory and can be written as [31]:

(4) $$S_{eff\;\;}=\frac{1}{\Delta n_{bulk}}\times \frac{p_{s}n_{s}-n_{i}^{2}}{\left(n_{s}+n_{i}\right)/S_{0p}+\left(p_{s}+n_{i}\right)/S_{0n}}$$

where $\Delta n_{bulk}$ is the excess carrier density, $p_{s}$ and $n_{s}$ are respectively the hole and electron concentration at the surface, $n_{i}$ is the intrinsic carrier concentration, and $S_{0p}$ and $S_{0n}$ are the *SRV* of holes and electrons, respectively. With variation of $S_{0n}$, $S_{0p}$, and $\tau_{bulk}$ as input simulation parameters, $\tau_{eff}$ is calculated by using Equation (3) and the carrier concentration is extracted from the simulation. The best fit of $\tau_{eff}$ as a function of carrier concentration between simulation and QSSPC lifetime measurement gives correct values of $S_{0n}$, $S_{0p}$, and $\tau_{bulk}$. The same values of $S_{0n}$ and $S_{0p}$ are used in the simulation.

**Figure 4:**
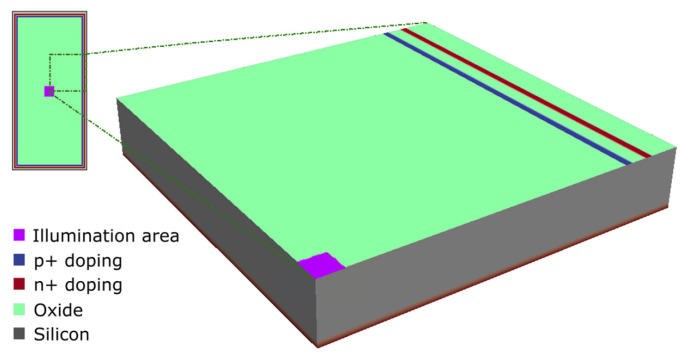
3D simulation structure of PQED photodiodes.

The second step is predicting the IQD of a photodiode, if manufactured by the given passivation recipe, by using a 3D simulation structure as shown in Figure 4 [9]. The structure consists of a doped silicon substrate, which is covered by a dielectric layer on the top and a doped layer on the bottom. Electrodes for electrical contacts are represented by n+ doping and p+ doping on top of the silicon substrate. Only 1/8 of the real device is used in the simulation due to computational limiting reasons, and the symmetric boundary conditions are applied to obtain the response of the whole photodiode. Besides the fabrication parameters which include fixed charge, doping concentration, etc., the fitted values $S_{0n}$, $S_{0p}$, and $\tau_{bulk}$ from the 2D simulation are used as input parameters in the 3D simulation model.

From the 3D simulation, the total surface recombination $R_{surf}$, total bulk recombination $R_{bulk}$, and total photon generation $G_{opt}$ are extracted and the *IQD* can be calculated by the following equation:

(5) $$IQD=\frac{R_{surf}+R_{bulk}}{G_{opt}}$$

### 3.2. Passivation Process and Sample Preparation

The SiN_x_ films are deposited at SINTEF MiNaLab using a conventional parallel plate capacitively coupled plasma (CCP) type PECVD reactor from SPTS [33]. The wafer is placed on a chuck and SiN_x_ films are deposited on the wafer by the reaction of gaseous precursors SiH_4_, NH_3,_ and N_2_. The plasma enhances the reaction rate, allowing the deposition to be performed at low temperatures. A SiN_x_ passivation process was established by using a pressure of 2000 mTorr, RF power of 40 W, chuck temperature of 350 °C, and electrode spacing of 20 mm. A gas flow rate of 60 sccm was used for NH_3_ and SiH_4_, and 3600 sccm was used for N_2_. The SiN_x_ deposition process developed originally (SiH_4_:NH_3_ gas flow ratio of 1:1) exhibits good passivation characteristics but too high optical absorption. The SiH_4_:NH_3_ gas flow ratio was then varied by changing the SiH_4_ flow rate while keeping all other process parameters the same to achieve the desired optical characteristics without degrading the passivation performance. Samples with SiH_4_:NH_3_ gas flow ratios of 1:3, 2:3, 2:1, and 3:1 were prepared for testing. Samples with SiN_x_ film thicknesses of 150 nm and 65 nm were prepared.

For our experiments, 500 µm thick, double-side polished FZ silicon wafers with <100> surface orientation from Topsil were used. The wafers were p-type with resistivity specification of 5000–12,000 Ω cm. The wafers were cleaned using the standard RCA process (NH_3_+HCl), followed by a diluted HF treatment prior to oxidation or SiN_x_ deposition. The wafers for lifetime measurements and ellipsometry measurements were passivated identically on both sides. The wafers used for fixed charge measurements were heavily implanted with boron on the backside for a good ohmic contact and passivated only on the front side. To make MIS structures, these wafers were then sputtered on both sides with 1.2 µm thick aluminum and the aluminum on the dielectric-coated side was patterned with a photolithography process followed by wet etching.

Some wafers were oxidized with a thin layer (6 nm) of thermally grown SiO_2_ prior to PECVD SiN_x_ deposition. Some of the oxidized samples were annealed prior to SiN_x_ deposition. One of the annealing processes is a gettering process which is employed in actual photodiode manufacturing to remove impurities and defects from silicon bulk. The wafers were also annealed in forming gas at 350 °C for 30 min, which is known to reduce the interface traps at the Si/SiO_2_ interface without affecting fixed charge density [34]. Annealing in forming gas was performed prior to SiN_x_ deposition as SiN_x_ is highly impermeable to H_2_ at such low temperatures.

### 3.3. Results

The original SiN_x_ deposition recipe developed using a SiH_4_:NH_3_ gas flow ratio of 1:1 was tested for SiN_x_ film thickness of 150 nm on unoxidized silicon. An effective lifetime of ~4 ms at an injection level Δ*n* of 6.5 × 10^14^ cm^−3^ and fixed charge density of ~3 × 10^12^ cm^−2^ were measured. However, ellipsometry measurements showed that this film has a high refractive index and absorbance with *n* and *k* values of 2.12 and 1.9 × 10^−3^ at a wavelength of 632 nm, respectively. The gas flow ratio of SiH_4_:NH_3_ was then varied to deposit SiN_x_ with different stoichiometric ratios while keeping all other parameters the same. Both the refractive index and extinction coefficient are strongly dependent on the stoichiometric ratio of SiN_x_ and increase with increasing Si content. The recipe with highest SiH_4_:NH_3_ flow ratio of 3:1, yielded an *n* value of 2.67 and *k* value of 4.8 × 10^−2^ at 632 nm. The recipe with SiH_4_:NH_3_ = 1:3 yielded the best optical properties with *n* = 1.84 and *k* = 0 at 632 nm. The lifetime and fixed charge measurements showed that the passivation properties of the SiN_x_ are not affected to any significant degree by the SiH_4_:NH_3_ gas flow ratio within the range of investigation. One would expect better passivation characteristics with increasing SiH_4_ flow in the deposition process due to the higher hydrogen content, which should provide more effective passivation of silicon dangling bonds [22]. However, the measurements showed that this was not the case, as probably even the recipe with lowest SiH_4_ flow was still providing enough hydrogen. These investigations showed that the recipe with SiH_4_:NH_3_ gas flow ratio of 1:3 leads to both optimal passivation and optical properties, with an effective lifetime of ~4 ms at injection level Δ*n* of 7.1 × 10^14^ cm^−3^, a fixed charge density of ~4 × 10^12^ cm^−2^, *n* = 1.84 and *k* = 0. This SiN_x_ deposition recipe was then chosen for further investigation and development.

**Figure 5:**
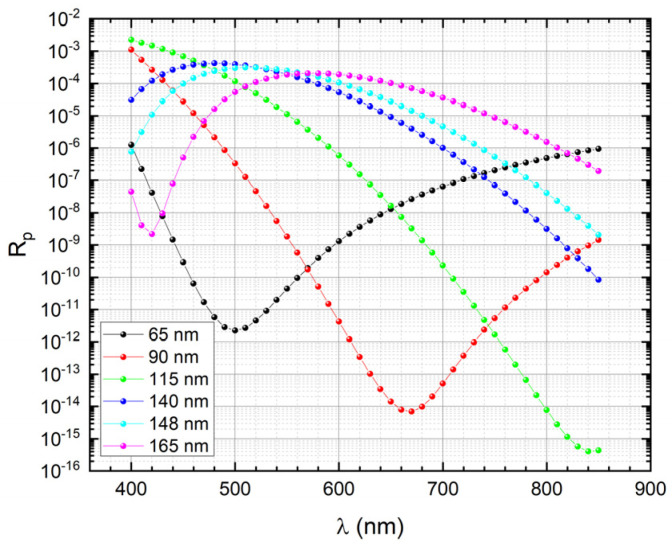
Simulated p—polarization reflectance as a function of wavelength for PQEDs mounted in trap configuration with an angle of 15° between the diodes. In this configuration the light beam undergoes 7 reflections: one at 0° degree and two reflections at 15°, 30°, and 45°. The reflectance is reported for six different thicknesses of the SiN_x_.

**Figure 6:**
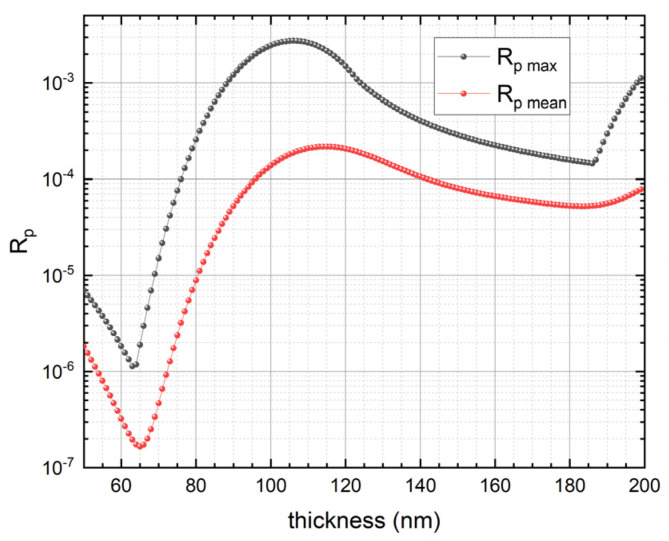
Maximum and mean values evaluated in the wavelength interval 400—850 nm of the p-polarization reflectance as a function of SiN_x_ thickness for PQEDs mounted in trap configuration with an angle of 15° between the diodes.

**Figure 7:**
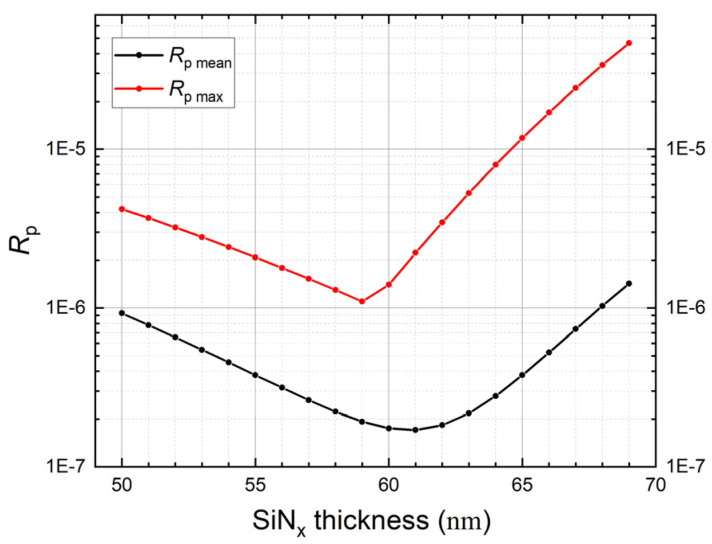
Maximum and mean values evaluated in the wavelength interval 400—850 nm of the p-polarization reflectance as a function of SiN_x_ thickness, when a buffer layer of 6 nm SiO_2_ is deposited before SiN_x_, for PQEDs mounted in trap configuration with an angle of 15° between the diodes.

The p-polarization reflectance of a 7-reflection trap configuration PQED consisting of two photodiodes passivated with the optimized SiN_x_ deposition recipe was simulated. Figure 5 shows the simulated reflectance as function of wavelength for different SiN_x_ thicknesses. Figure 6 shows the mean and maximum reflectance versus SiN_x_ thickness. These simulation results suggest an optimum SiN_x_ thickness of ~65 nm to minimize reflection losses. These simulations were also performed for a passivation dielectric stack including 6 nm thermally grown SiO_2_ underneath PECVD SiN_x_. Figure 7 indicates that the SiN_x_ thickness that leads to minimum reflectance becomes ~60 nm in presence of 6 nm SiO_2_.

The original SiN_x_ tests were performed on a film thickness of 150 nm. In order to confirm that the reduction of the film’s thickness to 60–65 nm does not degrade the passivation characteristics, new test samples with SiN_x_ thickness of 65 nm were then also prepared for testing in addition to samples with the original SiN_x_ thickness of 150 nm. This new batch also included test samples with SiN_x_ deposited on 6 nm thermally grown oxide and exposed to different annealing conditions. The process details of the samples are presented in Table 1.

**Figure 8:**
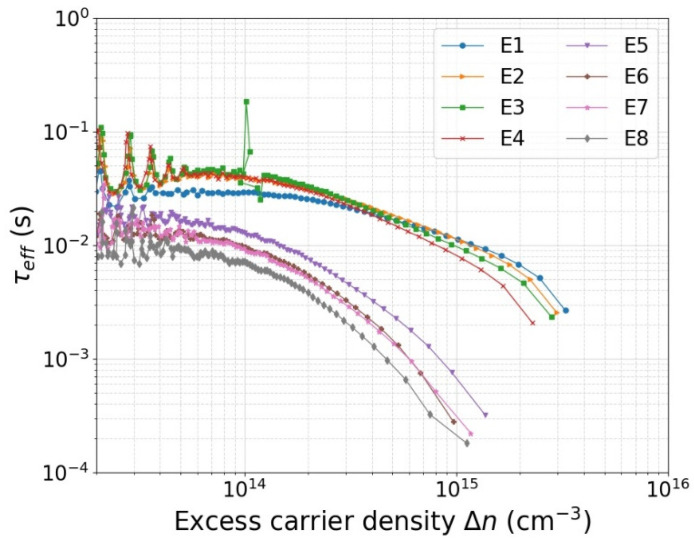
Effective lifetime *τ_eff_* vs. excess carrier density (Δ*n*) for samples prepared with passivation processes described in Table 1.

The QSSPC measurement results of all eight test samples that have gone through the processes described in Table 1 are shown in Figure 8. The wafers with a buffer layer of 6 nm thermally grown SiO_2_ underneath SiN_x_ clearly have a longer minority carrier lifetime, particularly at higher injection levels. The samples without SiO_2_ (*E5*, *E6*, *E7*, and *E8*) show a similar trend among each other. The samples that have thicker SiN_x_ and have gone through annealing in forming gas exhibit somewhat higher lifetime values over the entire range of injection density. This is in line with expectation, but the difference is quite small. The samples with a SiO_2_ layer (*E1*, *E2*, *E3*, and *E4*) also exhibit similar lifetime values among each other, but the trend as a function of injection level is slightly different at very low and very high injection levels.

**Figure 9:**
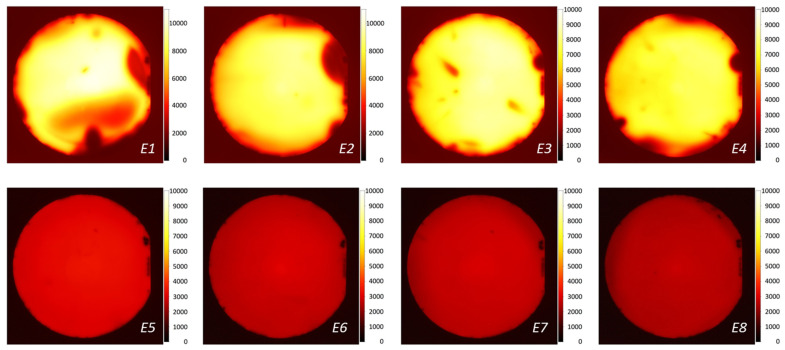
Photoluminescence (PL) lifetime images of samples prepared with the passivation processes described in Table 1. The color bar shows *τ_eff_* in µs.

The PL images showing the spatial variation of effective lifetime across the wafer were taken for all eight wafers. The PL images were calibrated using the measured QSSPC lifetime at injection levels shown in Table 2. The calibrated PL images are presented in Figure 9. The samples with SiO_2_/SiN_x_ passivation exhibit ~3 times higher effective lifetime compared with the samples with SiN_x_ passivation but inferior uniformity. The origin of this insignificant non-uniformity is not understood.

The C–V measurements have shown that reducing the SiN_x_ film thickness from 150 nm to 65 nm to minimize the reflectance losses does not affect the fixed charge density to any significant degree, eliminating processes *E1*, *E3*, *E5*, and *E7*. The gettering process after oxidation, which is an annealing process normally implemented to reduce silicon bulk impurities, has been proven to have no negative effect on the fixed charge density by comparing the C–V characteristics of samples *E2* and *E4*. Annealing in forming gas after SiN_x_ deposition is a process normally carried out in the end of photodiode fabrication for metal sintering, and skipping this process (namely, *E8*) has not shown any advantage, either. Therefore, passivation processes *E2* and *E6* were overall proven to be the most viable candidates among the group with thermal oxide and the group without thermal oxide, respectively.

**Figure 10:**
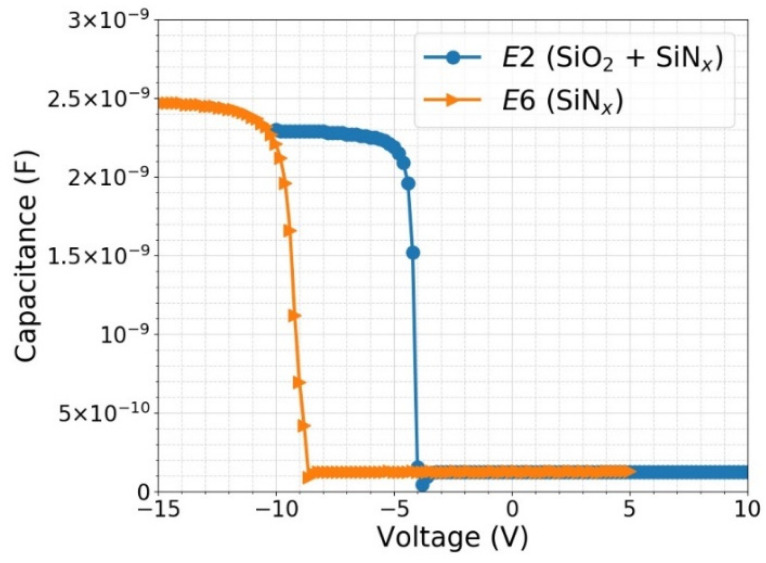
Capacitance—voltage (C—V) measurement results of MIS capacitors prepared with the passivation processes *E2* (6 nm SiO_2_ + 65 nm SiN_x_) and *E6* (65 nm SiN_x_) as described in Table 1, at a frequency of 1 kHz.

Figure 10 shows the measured C–V characteristics of MIS structures fabricated using passivation processes *E2* and *E6* at a frequency of 1 kHz. As mentioned in Section 3.1.2, the high resistivity of the silicon substrate retards the capacitance charging at high frequencies, leading to lower measured *C_acc_* than the theoretical value. Therefore, the measurements were performed at different frequencies to determine the optimum measurement frequency that yields reasonably accurate *C_acc_* with minimal noise, which was found to be 1 kHz. The fixed charge densities were determined from the C—V characteristics to be 1.3 × 10^12^ cm^−2^ and 4.0 × 10^12^ cm^−2^ for *E2* and *E6*, respectively. The passivation is expected to become gradually more dominated by SiO_2_ properties than SiN_x_ with increasing buffer SiO_2_ layer thickness. Therefore, a higher measured fixed charge density in bare SiN_x_ film as compared with a stack of SiO_2_ and SiN_x_ shouldn’t come as a surprise. Indeed, it has been reported that the overall fixed charge density in stack of Al_2_O_3_ (which is known to have negative fixed charge) with SiO_2_ turns positive as the interfacial SiO_2_ thickness becomes more than ~5 nm [35].

**Figure 11:**
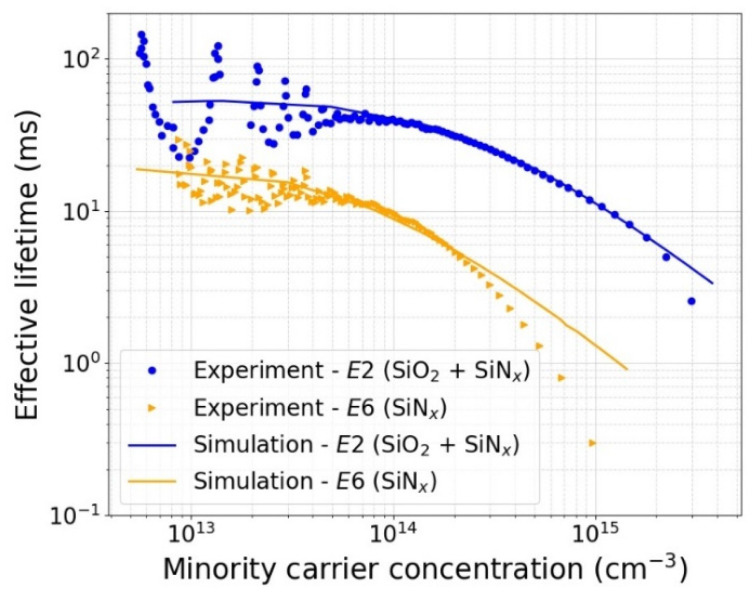
Injection dependent effective minority carrier lifetime *τ_eff_* (Δ*n*) of test samples passivated with processes *E2* (6 nm SiO_2_ + 65 nm SiN_x_) and *E6* (65 nm SiN_x_) as described in Table 1 with simulation fits to extract *SRV* and *τ_bulk_*.

Figure 11 shows the fit of the 2D simulation with the measured injection-dependent effective lifetime for passivation processes *E2* and *E6*. The simulated lifetime curves are obtained using the following fabrication and test parameters as inputs: substrate doping type (p-type), substrate doping concentration (2 × 10^12^ cm^−3^), substrate thickness (500 µm), and measured fixed charges (1.3 × 10^12^ cm^−2^ for *E2* and 4.0 × 10^12^ cm^−2^ for *E6*). The simulated lifetime is in an excellent agreement with the measured one up to an injection level of 2 × 10^15^ cm^−3^ for *E2*. For *E6*, on the other hand, the simulated lifetime fits the measured lifetime well only at lower injection levels and diverges from the measured one at injection levels above 2 × 10^14^ cm^−3^. The bulk lifetime and surface recombination velocities obtained from these fits are reported in Table 3.

### 3.4. Predicted Photodiode Responsivity

**Figure 12:**
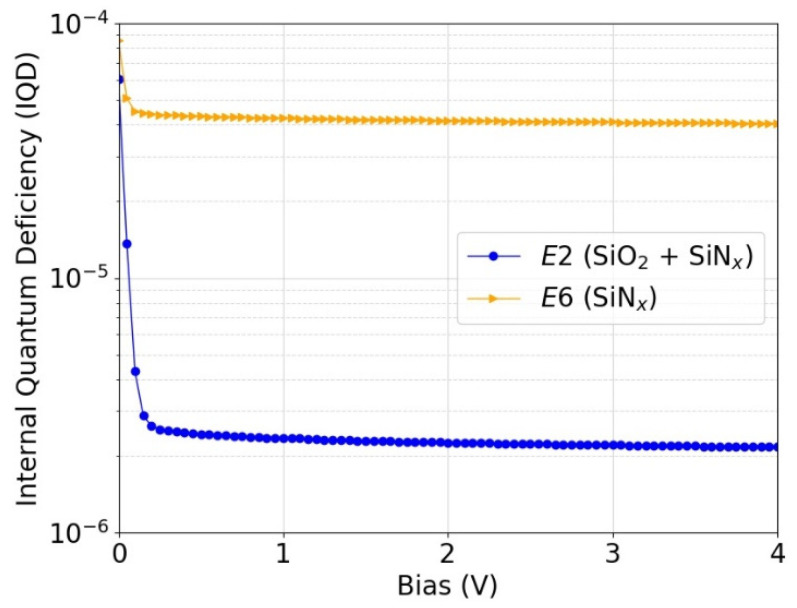
Simulated IQD as a function of reverse bias voltage for p-type inversion-layer photodiode that would be fabricated with passivation *E2* (6 nm SiO_2_ + 65 nm SiN_x_) and *E6* (65 nm SiN_x_) as described in Table 1. The simulations were performed at a wavelength of 488 nm.

**Figure 13:**
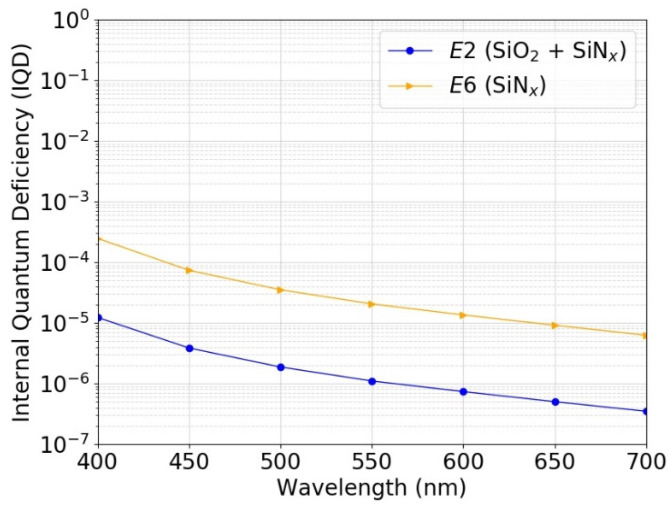
Simulated IQD as a function of wavelength for p-type inversion-layer photodiode that would be fabricated with passivation *E2* (6 nm SiO_2_ + 65 nm SiN_x_) and *E6* (65 nm SiN_x_) as described in Table 1. The simulations were performed at a reverse bias voltage of 5 V.

Using the extracted SRV and bulk lifetime (*τ_bulk_*), the IQD of a photodiode that would be made with the corresponding passivation was simulated using 3D simulation models. Figure 12 shows the simulated IQD as a function of reverse bias voltage for a photodiode passivated with the process *E2* or *E6*. Figure 13 shows the simulated IQD as a function of wavelength at reverse bias voltage of 5 V. The passivation process *E2* (SiO_2_ + SiN_x_) is estimated to lead to IQDs as low as ~2 ppm at 488 nm and to more than a factor of 10 lower IQD compared with the passivation process *E6* (SiN_x_).

## 4. Photodiode Design and Fabrication

Photodiodes of active area size of 11 mm × 11 mm, 11 mm × 14 mm, and 11 mm × 22 mm were included in the wafer layout. The photodiode design matches the cross-sectional sketch depicted in Figure 1 and includes n+ ring for electrical contact to the active area, p-stop ring to isolate n+ electrodes, n+ guard ring to shield the active area from the sensor edge effects, and p+ ring around the physical edge of the photodiode for electrical contact to the p+ electrode from the top side of the photodiode.

**Figure 14:**
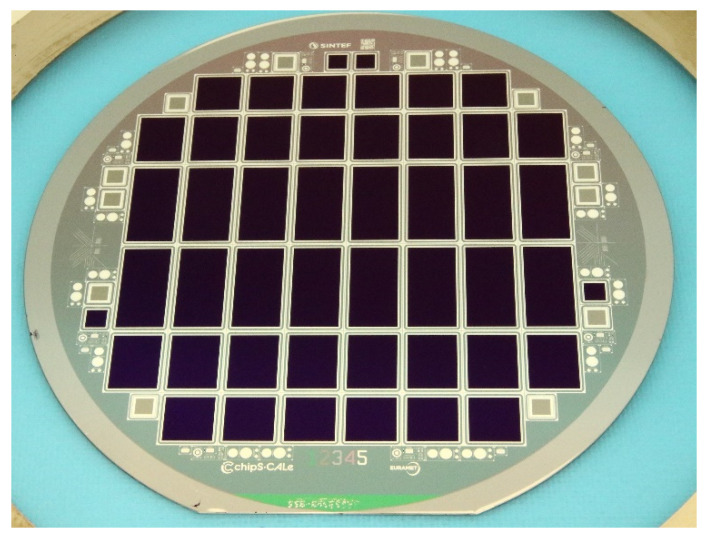
Completed 6-inch wafer of p-type inversion layer photodiodes passivated with SiO_2_/SiN_x_.

The photodiode fabrication was carried out in SINTEF MiNaLab cleanroom facilities. The same wafer material as the one used for the passivation tests discussed in the previous section was used for photodiode fabrication (500 µm thick, p-type FZ wafers with resistivity range of 5000–12,000 Ωcm and <100> surface orientation). The wafers were first thermally oxidized to grow thick SiO_2_ that is used as field oxide throughout the processing. The wafers were processed with 5 photomask layers that were used to define the areas of p+ implantation, n+ implantation, active area passivation dielectric, contact holes through SiO_2_, and metallization. The p+ and n+ electrodes were made with boron and phosphorous implantation, respectively. On most wafers, the active area passivation was done using the process optimized in the previous section to minimize reflection losses and IQD, namely, process *E2* (6 nm SiO_2_ + 65 nm SiN_x_). On a couple of wafers, process *E6* (65 nm SiN_x_) was implemented for comparison and validation of the test and simulation results. The wafers were metallized with 1.2 µm aluminum on both sides. Figure 14 shows a picture of a completed wafer of photodiodes.

The I–V characteristics of all photodiodes were measured at room temperature at wafer level. The measurements were carried out by using the biasing scheme shown in Figure 1. The measurement results show some variation across the wafer and between wafers, with typical active area leakage current values in the range of 1–5 nA/cm^2^ at reverse bias voltage of −20 V.

## 5. PQED Assembly and Test Results

### 5.1. PQED Assembly

**Figure 15:**
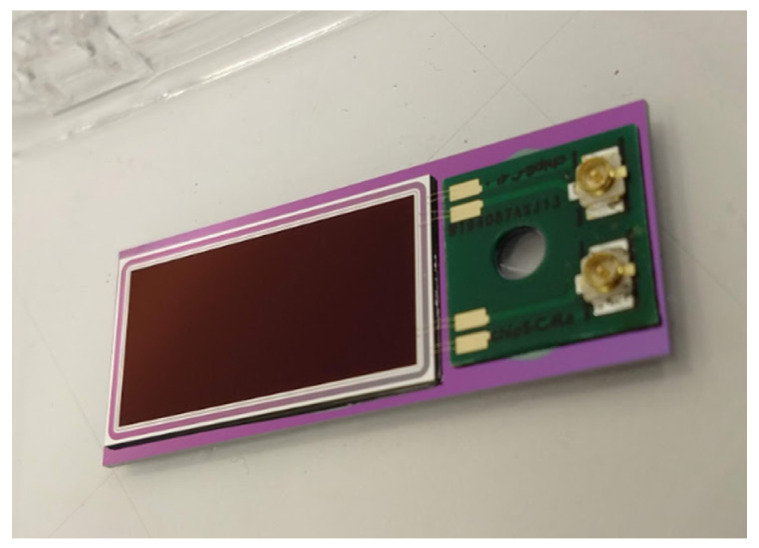
Photodiode packaging: photodiode and PCB mounted on silicon carrier.

The photodiodes with physical dimensions of 13 mm × 24 mm (active area dimensions of 11 mm × 22 mm) were adhesively bonded to silicon carriers having dimensions of 15 mm × 38 mm (Figure 15). On the remaining space on each carrier, a 12 mm × 12 mm printed circuit board (PCB) was glued. The PCB and chip carrier have 3 mm diameter holes for screw assembly into PQED mechanics. Wire bonds were made between the photodiode and the PCB using an Au ball-wedge bonder with 17.5 µm wire diameter. U.FL connectors soldered to PCB enables electrical connections to external instruments.

Both the PCB and photodiode were aligned and placed on the carrier using a Finetech pico die bonder. Stycast 1266 epoxy was used for all adhesive bonds, which, due to its low viscosity, easily spreads out during bonding and forms thin layers, ensuring that the photodiode is parallel to the carrier. The PCB metallization was 35 µm copper with electroless nickel immersion gold (ENIG) coating in order to facilitate wire bonding.

The packaged photodiodes were mounted in a mechanical wedge trap structure to form windowless seven-reflection PQEDs (Figure 2). The mechanical housings were purged with a constant flow of N_2_ to prevent dust particles from entering inside the detector. Alignment of the PQEDs was carried out with a laser beam at 405 nm on the same optical path as in the measurements at 488 nm. Reflection from the PQED is visible to the eye at 405 nm but not at 488 nm for laser beam power of less than 1 mW.

### 5.2. IQD Measurements and Comparison with Simulated Response

**Figure 16:**
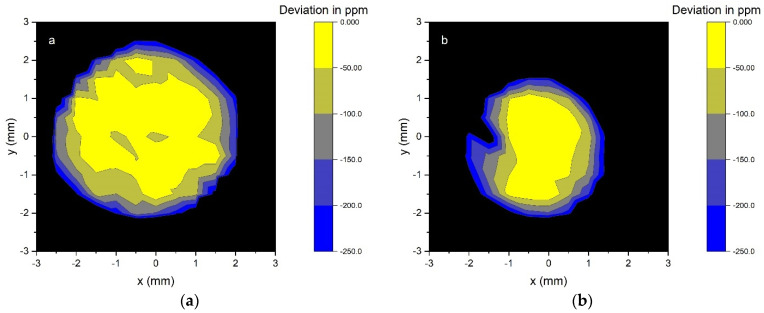
Spatial uniformity of optical power responsivity of the PQEDs with SiO_2_/SiN_x_ stack photodiodes *P18-55-45* (**a**) and *P18-54-44* (**b**).

Spatial uniformity of the PQEDs was measured at 488 nm with a 1/*e*^2^ beam diameter of 2.4 mm and power of 100 µW to 150 µW (Figure 16). All PQEDs were measured with 5 V reverse bias. The responsivity of the PQEDs is constant within approximately 50 ppm peak-to-peak over the central area with a diameter of 1 mm to 3 mm.

The absolute responsivity was determined at the center of each PQED against a reference PQED with a predicted external quantum deficiency of 37_−9_ ^+140^ ppm [5,7,36], where the asymmetric uncertainty limits correspond to 95% confidence level. The responsivity estimates of the reference PQED are validated via another PQED with measurements against a cryogenic radiometer [6]. The temporal stability of the reference PQED was confirmed by responsivity measurements over a period of almost ten years [37].

The PQED with purely SiN_x_ passivation layer had 9 ppm higher responsivity than the reference PQED, while the PQEDs made of SiO_2_/SiN_x_ stack photodiodes *P18-55-45* and *P18-54-44* had 36 ppm and 16 ppm higher responsivity than the reference PQED, respectively. The standard uncertainty of 30 ppm of the above results is determined by the photocurrent ratio measurement and spatial uniformity of the detectors (Figure 16). After taking into account the negligible reflectance loss of SiN_x_ and SiO_2_/SiN_x_ stack PQEDs, these results translate into measured IQD values of 28 ppm (SiN_x_), 1 ppm (*P18-55-45*), and 21 ppm (*P18-54-44*). This confirms that the manufactured photodiodes with the SiO_2_/SiN_x_ stack have a very low IQD.

The measured IQD values of the PQEDs are consistent with the predicted responsivity from the lifetime measurements of the passivation test samples given in Figure 12 and Figure 13. The measured IQD values are so small that their deviation from zero is beyond the usual capabilities of radiometric measurements and substantially more work is needed to validate the predicted charge carrier losses of the SiO_2_/SiN_x_ stack PQEDs at such uncertainty levels. The evidence we have managed to achieve supports the lifetime curve fit prediction method and shows that the photodiodes have a record low external quantum deficiency.

## 6. Conclusions

We thoroughly investigated and optimized PECVD SiN_x_ passivation with and without a buffer layer of 6 nm thermally grown SiO_2_ for surface recombination and optical losses to develop PQEDs with negligible quantum deficiency based on p-type-induced-junction photodiodes. We demonstrated excellent passivation characteristics (*Q_f_* > 1 × 10^12^ cm^−2^ and *SRV* ≈ 1500 cm/s) and optical characteristics (*n* = 1.84 at wavelength of 632 nm and *k* = 0 for the whole range between 400 nm to 850 nm) with an optimized stack of thermal SiO_2_ and PECVD SiN_x_. PQEDs assembled of photodiodes with optimized passivation were tested against a reference PQED and showed record high quantum efficiencies with IQDs down to 1 ppm with 30 ppm measurement uncertainty. Within the measurement uncertainties, the efficiency results are in line with the predictions of the simulations based on the passivation and optical parameters extracted from test samples.

## Figures and Tables

**Table 1:** Process description of test samples.

Sample ID	Thermally Grown SiO_2_	Gettering After Oxidation	Annealing in Forming Gas after Oxidation	PECVD SiN_x_	Annealing in Forming Gas after PECVD SiN_x_ Deposition
*E1*	6 nm	Yes	Yes	150 nm	Yes
*E2*	6 nm	Yes	Yes	65 nm	Yes
*E3*	6 nm	No	Yes	150 nm	Yes
*E4*	6 nm	No	No	65 nm	Yes
*E5*	-	N/A	N/A	150 nm	Yes
*E6*	-	N/A	N/A	65 nm	Yes
*E7*	-	N/A	N/A	150 nm	No
*E8*	-	N/A	N/A	65 nm	No

**Table 2:** Injection level and corresponding lifetime used for calibration of PL images for different samples.

Sample ID	Δ*n* (cm^−3^)	*τ_eff_* (ms)
*E1*	1.8 × 10^15^	10.9
*E2*	1.7× 10^15^	10.2
*E3*	1.6 × 10^15^	9.4
*E4*	1.5 × 10^15^	8.6
*E5*	6.5 × 10^14^	3.9
*E6*	5.9 × 10^14^	3.5
*E7*	5.7 × 10^14^	3.4
*E8*	5.3 × 10^14^	3.1

**Table 3:** Extracted bulk lifetime and surface recombination velocities for passivation processes *E2* and *E6*.

Sample ID	*E2*	*E6*
Structure	SiN_x_/SiO_2_/Si	SiN_x_/Si
Fixed charge density *Q_f_*	1.3 × 10^12^ cm^−2^	4 × 10^12^ cm^−2^
Simulation bulk lifetime (*τ_bulk_*)	30 ms	12 ms
Simulation SRV (*S*_0*n*_, *S*_0*p*_)	1.5 × 10^3^ cm/s	6.0 × 10^4^ cm/s

## Data Availability

Data available in a publicly accessible repository. The data presented in this study are openly available in Zenodo at 10.5281/zenodo.5720711.
